# Supplementary material for: Data-driven discovery of electrocatalysts for CO2 reduction using active motifs-based machine learning
Source: Nat Commun. 2023 Nov 11;14:7303. doi: 10.1038/s41467-023-43118-0 (PMC10640609; doi:10.1038/s41467-023-43118-0)
Supplement: Supplementary file 1 — Supplementary Information [file 41467_2023_43118_MOESM1_ESM.pdf]

Supplementary Information for

"Data-driven Discovery of Electrocatalysts for CO_2 Reduction using Active Motifs-based Machine Learning"

Dong Hyeon Mok[1,#], Hong Li[2,#], Guiru Zhang[2], Chaehyeon Lee[1], Kun Jiang[2,] and Seoin Back[1,*]*

[1]Department of Chemical and Biomolecular Engineering, Institute of Emergent Materials, Sogang University, Seoul 04107, Republic of Korea

[2]Interdisciplinary Research Center, School of Mechanical Engineering, Shanghai Jiao Tong University, Shanghai 200240, China.

[#]These authors contributed equally to this work.

Corresponding Authors

E-mail: seoin0226@gmail.com (S. B.), kunjiang@sjtu.edu.cn (K. J.)

Supplementary Note 1: DSTAR-based High-throughput Virtual Screening Pipeline

As shown in **Figure S1**, DSTAR-based high-throughput screening begins by (i) converting all surfaces in the dataset into active motif-based representations, which are then used as fingerprints to train regression models. The representation includes positional information of the active motif, which is divided into three sites: the first nearest neighbor (FNN) atoms of the adsorbates, the second nearest neighbor atoms in the surface layer (SNN_{same}), and the sublayer (SNN_{sub}). Then, it incorporates their weighted average elemental properties (Atomic number, Block, Ionic radius, Oxidation state, Electronegativity, Row, Group, Thermal conductivity, Boiling point, Melting point, First ionization energy) and the number of atoms of each site. (ii) The regression model is trained using the active motif representations and their corresponding binding energy. (iii) All active motif representations are converted into prototypes of active motifs that only contains positional information without any elemental details. This is to remove duplicates and collect unique active motifs. (iv) To expand the chemical space, new elemental combinations that are outside the original domain are inserted into the prototype of unique active motifs, resulting in a new set of active motif representations. The number of active motifs generated per elemental combination is the same as the number of prototypes of unique active motifs. (v) The trained regression model predicts the binding energies of the new set of active motifs. The predicted binding energies are plotted on a 3D selectivity map to calculate the product selectivity of a specific elemental combination.

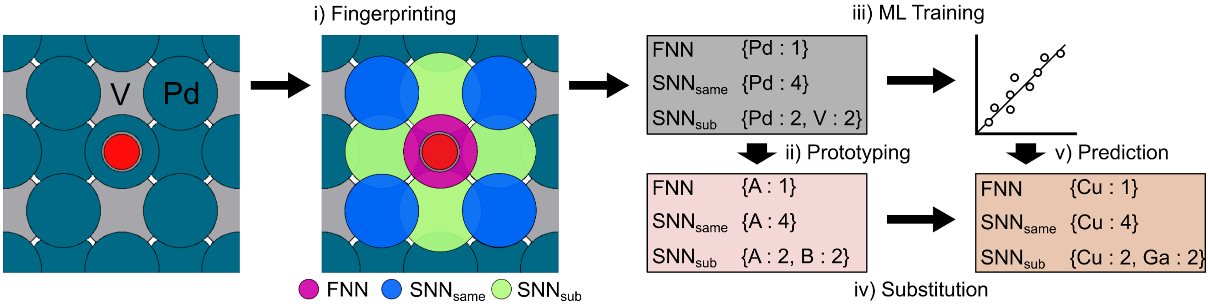

Figure S1. Schematics of high-throughput virtual screening using DSTAR.

Supplementary Note 2: Reaction Mechanism of CO_2RR

The initial protonation step of CO_2 reduction can proceed through two pathways; formation of an O-H bond to produce COOH* (reaction (i)) and formation of a C-H bond to produce HCOO* species (reaction (ii)).

$$CO_2\,(g) + H^+ + e^- + * \rightarrow COOH* \quad \text{(i)}$$

$$CO_2\,(g) + H* \rightarrow HCOO* \quad \text{(ii)}$$

Reaction (i) results in the production of CO*/CO (g), which is a key intermediate that can be further reduced to produce C_1 or C_{2+} products, while reaction (ii) typically leads to the production of HCOOH production. We note that reaction (ii) is a chemical step which requires a prior surface protonation stp (reaction (iii)), so-called the Volmer step.

$$H^+ + e^- + * \rightarrow H* \quad \text{(iii)}$$

We will also consider the possibility of surface poisoning by OH* when the binding energy of OH* is strong enough to inhibit its protonation to water (reaction (iv)).

$$OH* + H^+ + e^- + * \rightarrow H_2O + * \quad \text{(iv)}$$

The boundary conditions for the competition between HCOO* vs COOH* pathways, as well as the two additional reactions that determine surface adsorption states, can be expressed by following equations:

$$\Delta G_{rxn}^{(i)} = \Delta G_{rxn}^{(ii)} \quad (1)$$

$$\Delta G_{rxn}^{(iii)} = 0 \text{ eV} \quad (2)$$

$$\Delta G_{rxn}^{(iv)} = 0 \text{ eV} \quad (3)$$

After the production of CO* following the COOH* pathway, protonation of CO* can proceed to two possible intermediates, either CHO* or COH*. In this study, we referred to the results of energetic and kinetic investigations on both CO* protonation pathways performed by

Tang et al.[1] These investigations suggested that the COH* pathway (reaction (v)) is more energetically accessible than the CHO* pathway due to the kinetically locked nature of the CHO* pathway at the negative potential. As the direct protonation of CO* to CHO* (CO* + H^+ + e^- → CHO*) is significantly sluggish compared to COH* formation, CHO* formation primarily relies on the thermal coupling of CO* and adsorbed H* (CO* + H^* → COH*), requiring the preceding Volmer reaction (H^+ + e^- + * → H*). However, the coupling barrier of CO* and H* remains independent of the applied potential, rendering the COH* pathway more favorable at the operating potential regions. Consequently, in this study, we will exclusively focus on the COH* pathway for the generation of C_{1+} products.

CO* + H^+ + e^- → COH* ($G_{CO*} < 0$) (v)

CO(g) + H^+ + e^- + * → COH* ($G_{CO*} > 0$)

For the further reduction of COH* toward C_{1+} products, the protonation of CO* to form COH* is known to be the rate-determining step[2]. Thus, we used the reaction Gibbs energy of CO* protonation as a boundary condition to determine its further protonation. The criterion was set as follows.

$$\Delta G_{rxn}^{(v)} = 0.75 \text{ eV} \quad (4)$$

The reaction Gibbs energy was used as the criterion instead of the activation energy, since the former was found be slightly lower than the latter for Cu (100), and much more efficient to calculate.[1]

To establish boundary conditions for CO_2RR against hydrogen evolution reaction (HER), we refer to the theoretical results by Tang et al. regarding CH_x* formation. It was suggested that the protonation of CH* to CH_2* is the most unfavorable step after the electroreduction of CO* to C*. The competition between CH* + H^+ + e^- → CH_2* *vs.* * + H^+ + e^- → H* is identical to the potential independent condition as follows:

$$CH^* + H^* \rightarrow CH_2^* + * \quad \text{(vi)}$$

Further protonation of H*, also known as Heyrovsky reaction, produces H_2, (reaction (vii)).

$$H^* + H^+ + e^- \rightarrow H_2 + * \quad \text{(vii)}$$

Thus, two boundary conditions for determining HER selective region are

$$\Delta G_{rxn}^{(vi)} = 0 \text{ eV} \quad (5)$$

$$\Delta G_{rxn}^{(vii)} = 0 \text{ eV} \quad (6)$$

The thermodynamic boundary conditions are represented by three binding energy descriptors (ΔE_{CO^*}, ΔE_{H^*} and ΔE_{OH^*}) using linear scaling relationships between these descriptors and other intermediates of CO_2RR. We note that the applied potential term, *eU*, in boundary conditions demonstrates its potential-dependency. The details of the scaling lines, free energy diagram, boundary conditions represented by energetic descriptors and applied potential are enumerated **Figure S3**, **Figure S4** and **Table S3**, respectively.

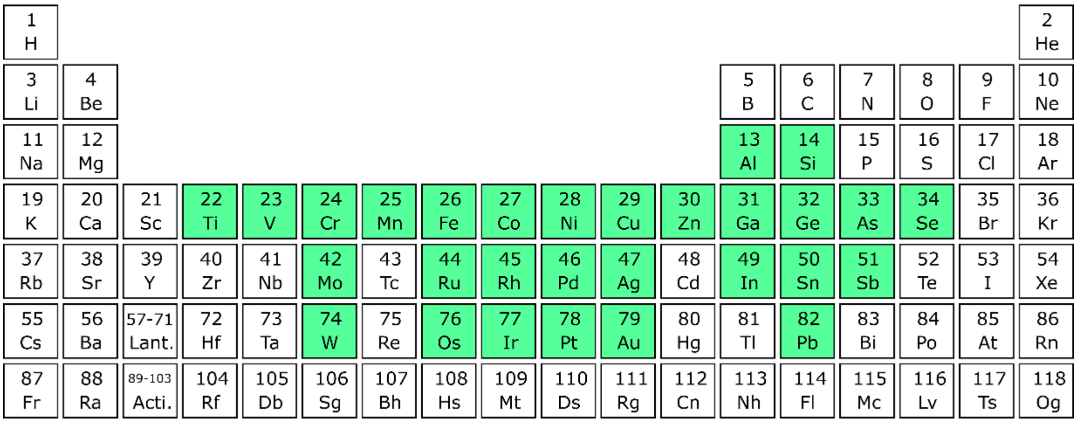

Figure S2. Periodic table highlighting the elements used in this work.

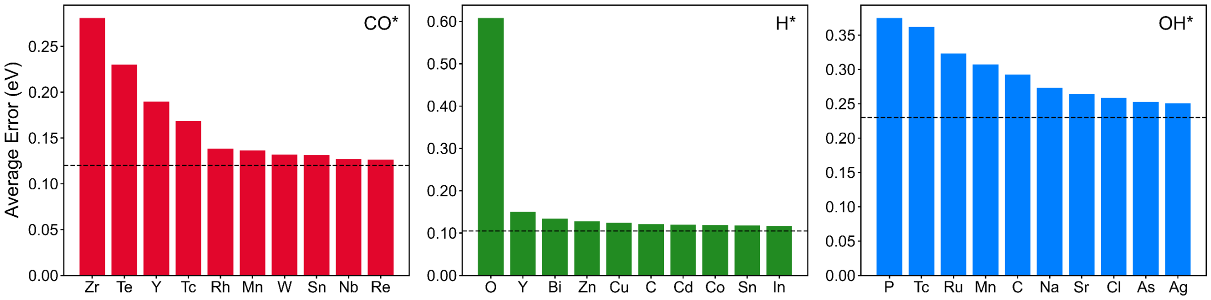

Figure S3. Average binding energy prediction error of CO* (Red), H* (Green) and OH* (Blue) when specific elements are included in local surface. Dashed line indicates MAE of ML for each adsorbate. A large error means that the model is likely to incorrectly predict the binding energy of surface containing the corresponding element.

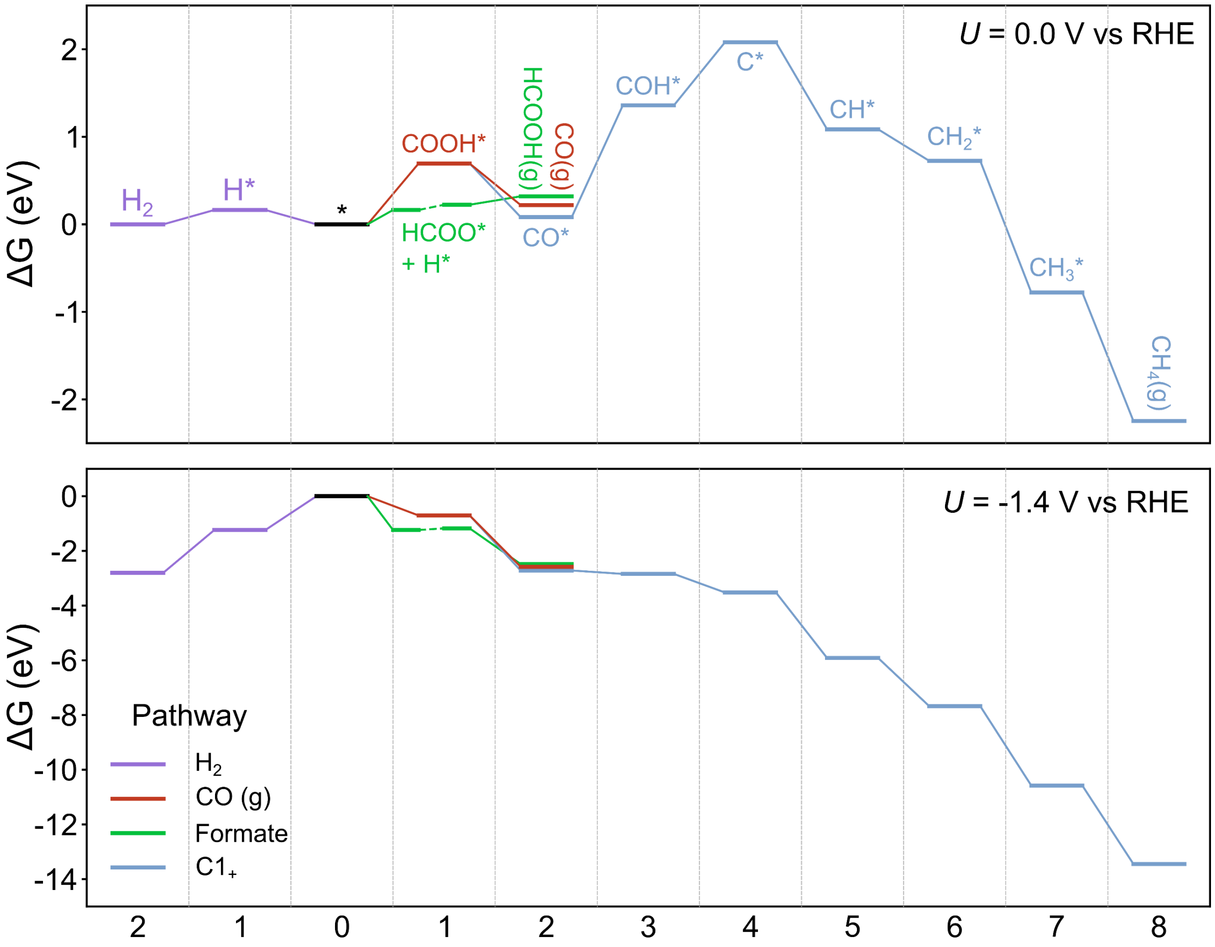

Figure S4. The Gibbs free energy diagram of CO2RR on Cu (111) at U = 0.0 V and -1.4 V vs. RHE. The pathways for four products are shown: H_2 (Purple), HCOOH (Green), CO (Red) and CH_4 (Blue)

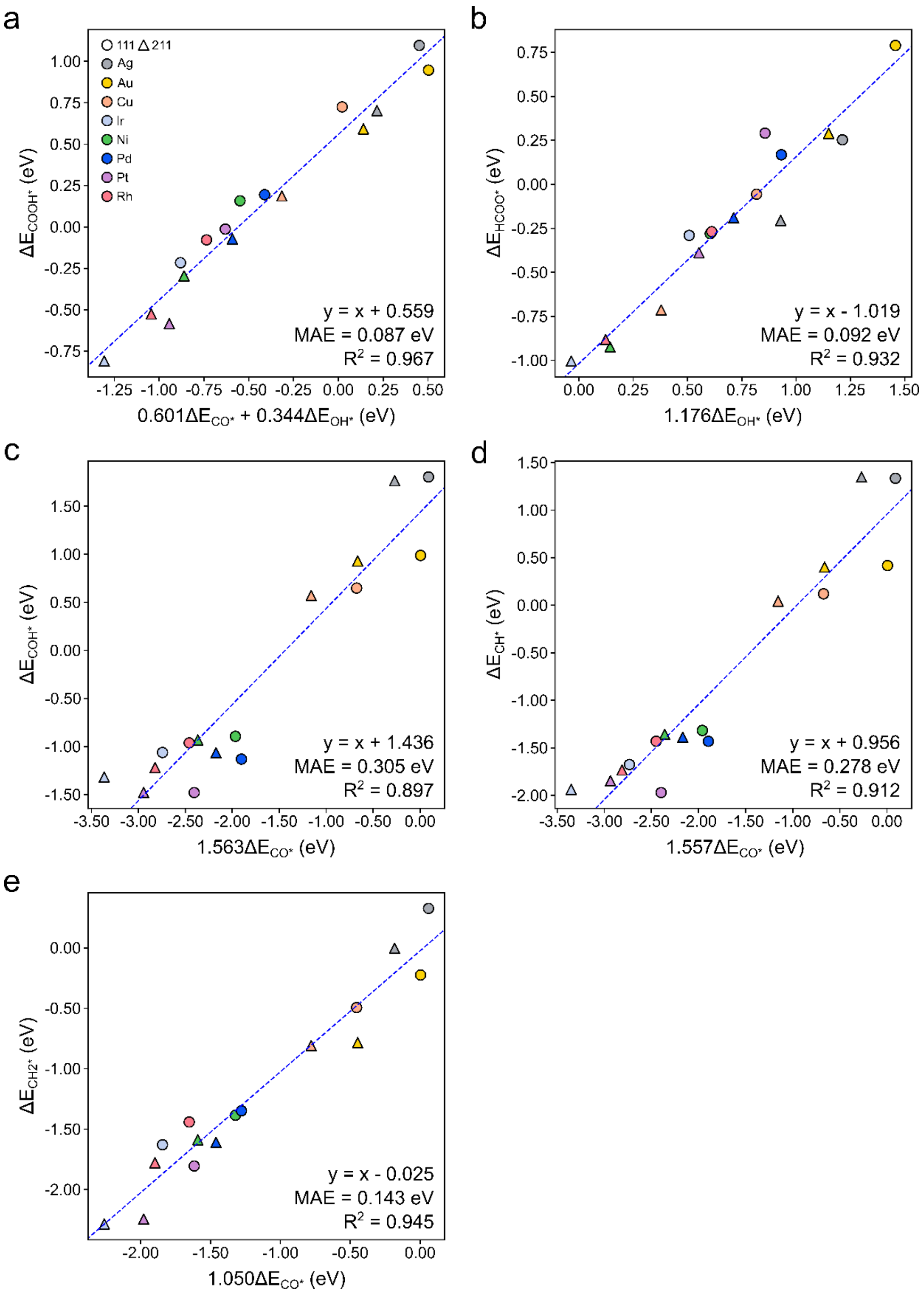

Figure S5. The scaling relations between adsorption energies of different reaction intermediates and combinations of descriptors (ΔE_{CO*} and ΔE_{OH*}), along with their corresponding linear equations, mean absolute error (MAE) and R^2 scores. (a) ΔE_{COOH*}, (b) ΔE_{HCOO*}, (c) ΔE_{COH*}, (d) ΔE_{CH*} and (e) ΔE_{CH2*}.

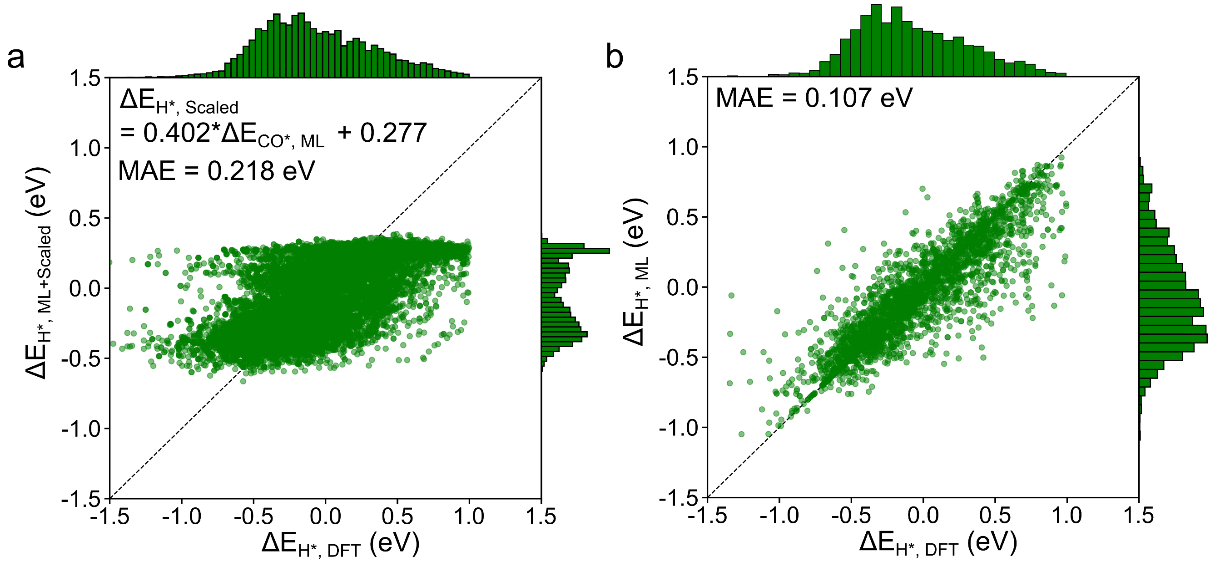

Figure S6. Parity plots of (a) the estimated ($\Delta E_{H^*,ML+Scaled}$) and DFT calculated ($\Delta E_{H^*,DFT}$) H* binding energy, and (b) directly predicted ($\Delta E_{H^*,ML}$) and DFT calculated ($\Delta E_{H^*,DFT}$) H* binding energy. The estimation is based on the ML predicted CO* binding energy ($\Delta E_{CO^*,ML}$) and the scaling relation between ΔE_{CO^*} and ΔE_{H^*}.

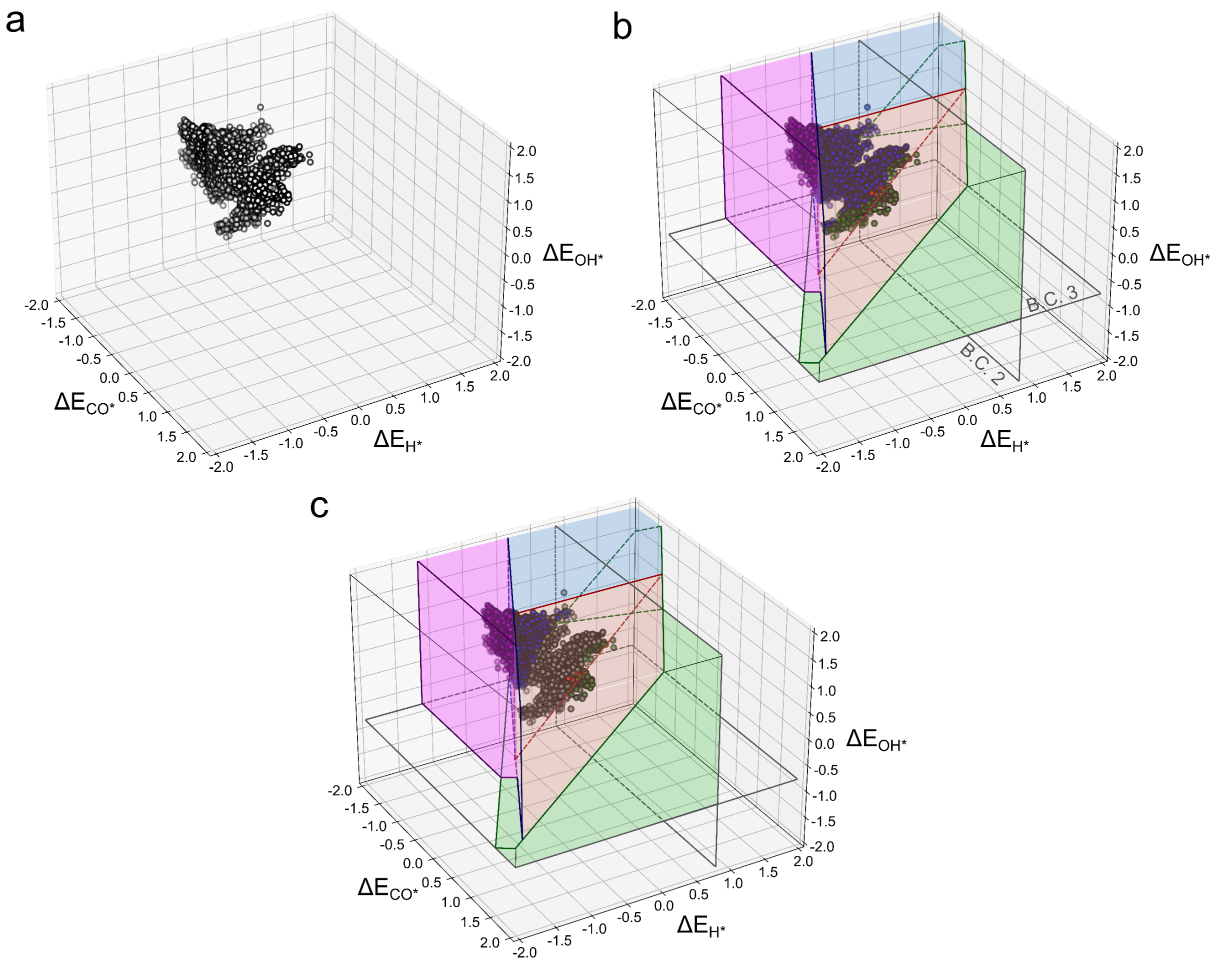

Figure S7. The example of utilizing selectivity map. (a) All unique active motifs are scattered in the map. Each point is converted into a cuboid volume, considering MAE of ML predictions. (b) The thermodynamic boundary conditions are applied and the volume in the map that each active motif occupies is identified. (c) The active motifs that do not satisfy the reaction energy condition ($\Delta G_{\mathrm{MAX}} < 0$ eV at the applied potential) are removed (grey). The remaining active motifs are used to calculate productivity of each product.

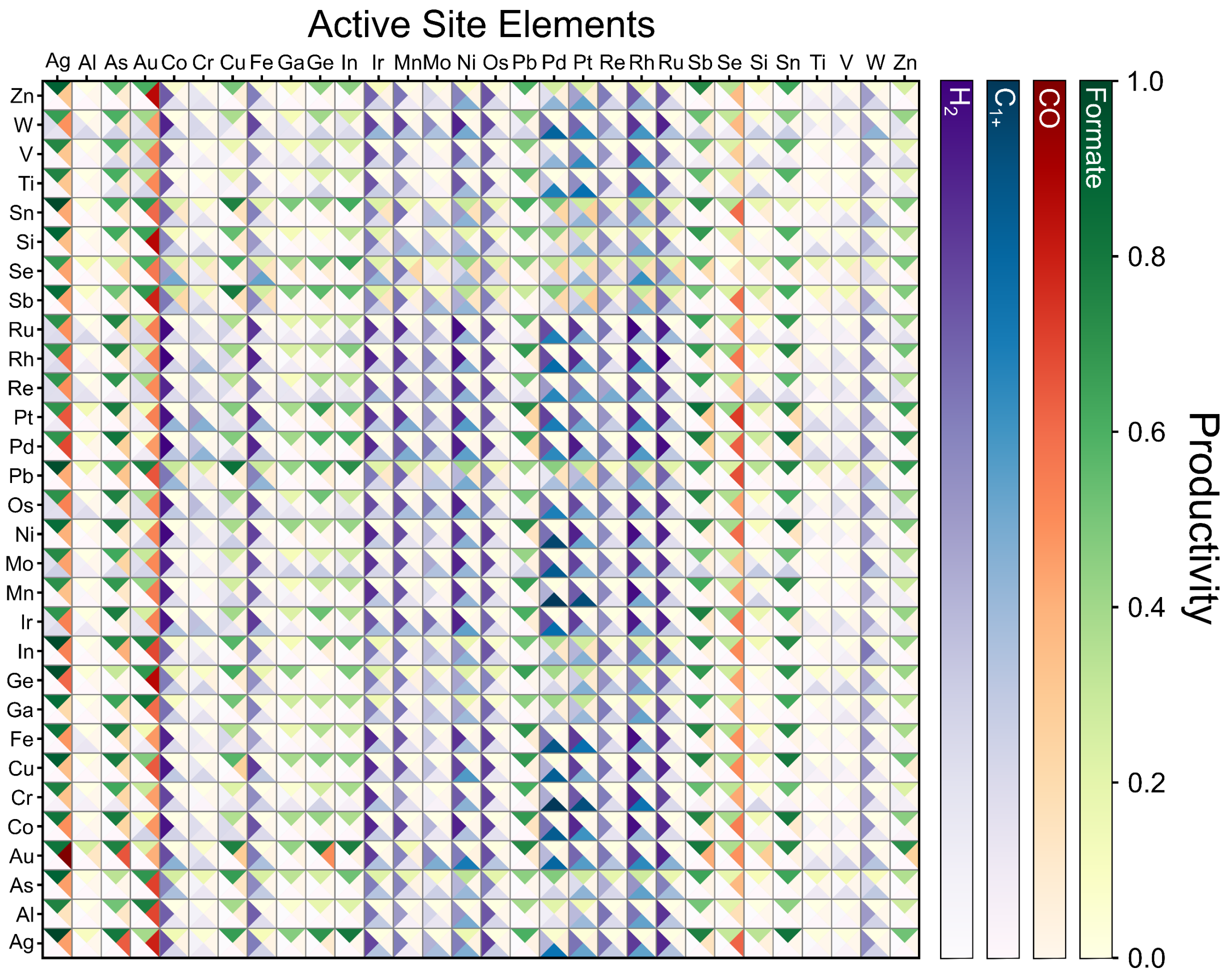

Figure S8. Productivity heatmap visualizing the normalized productivity; Formate (green, top), CO (red, right), C_{1+} (blue, bottom), H_2 (purple, left) at $U = -1.0\ V_{RHE}$.

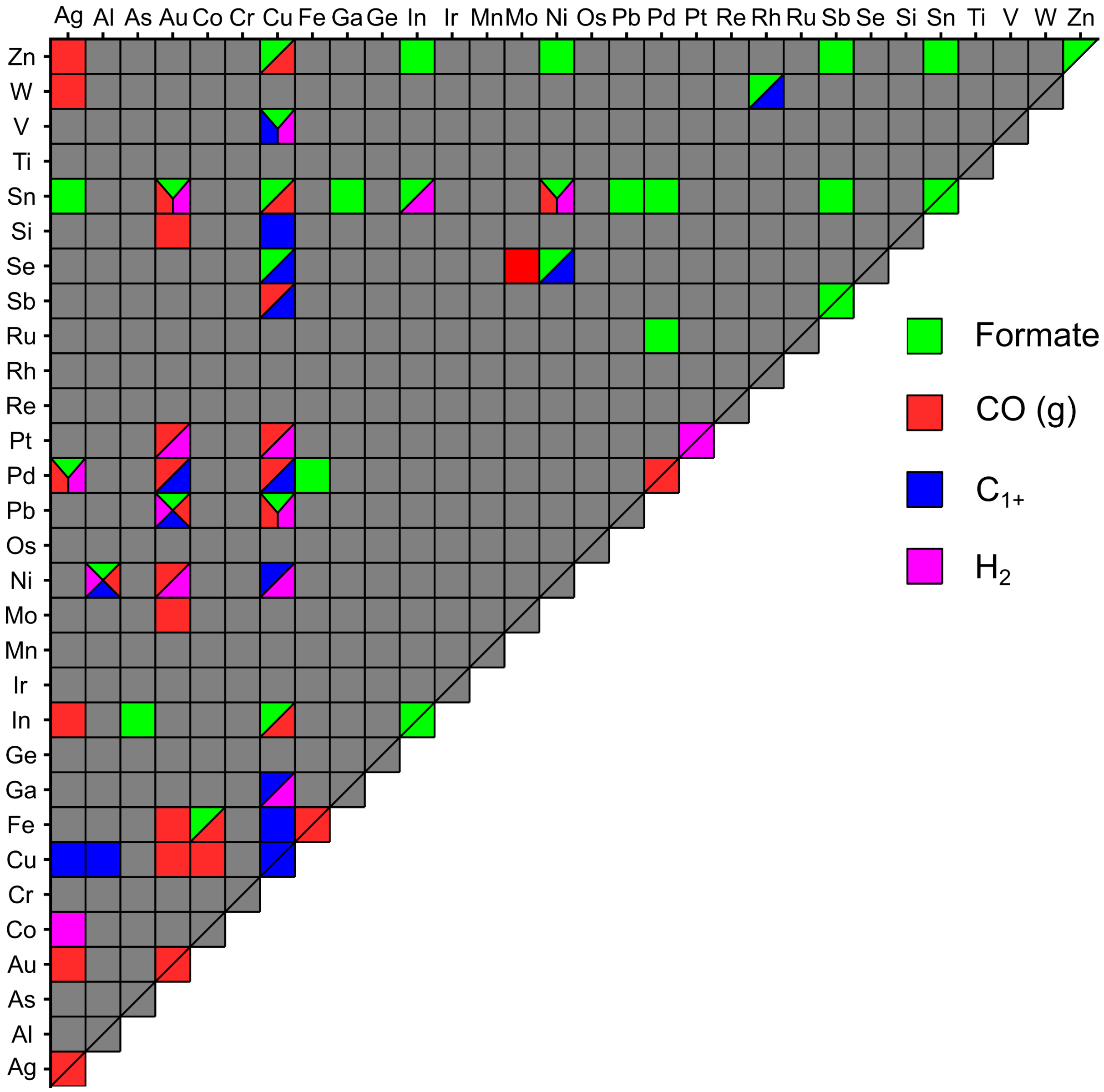

Figure S9. Heatmap visualizing electrochemical CO_2RR products (Formate : Green, CO : Red, C_{1+} : Blue, H_2 : Purple, Unknown : Gray) of metal alloys reported in literatures when searching for the keyword: “Carbon dioxide reduction catalyst” in Web of Science.

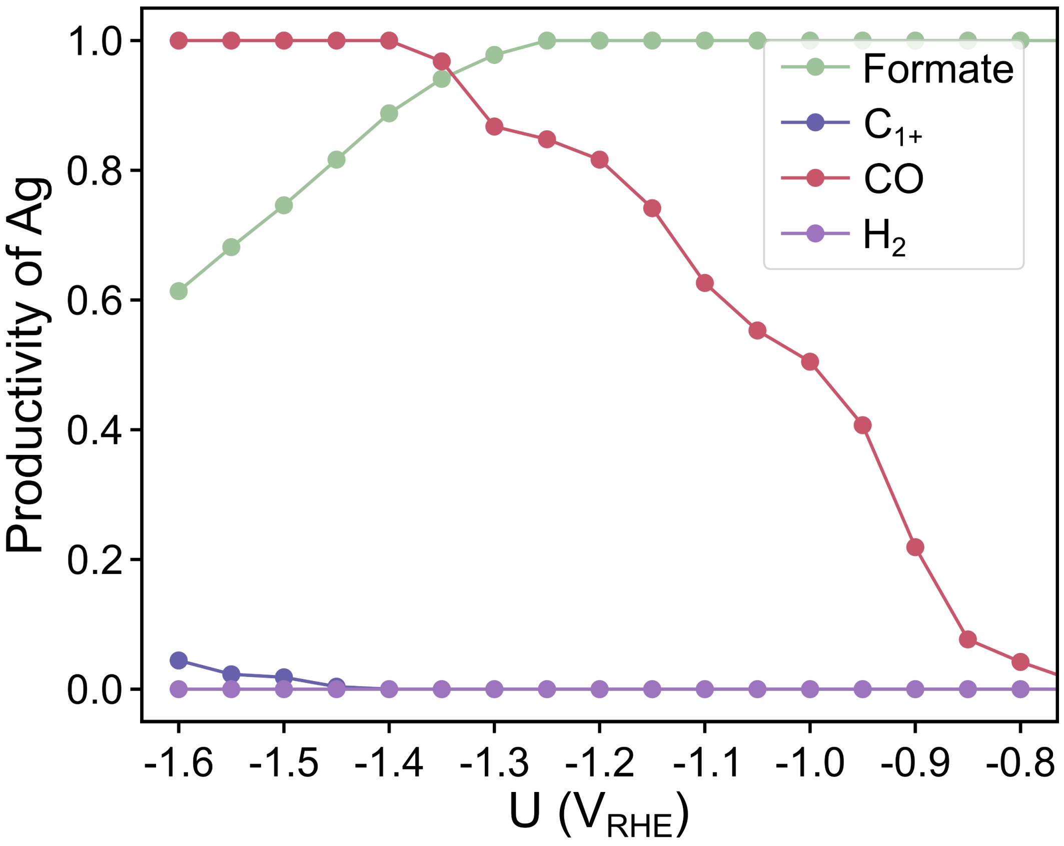

Figure S10. ML-predicted product selectivity of pure Ag. At potentials more positive than −1.3 V_{RHE}, the product selectivity for formate is dominant.

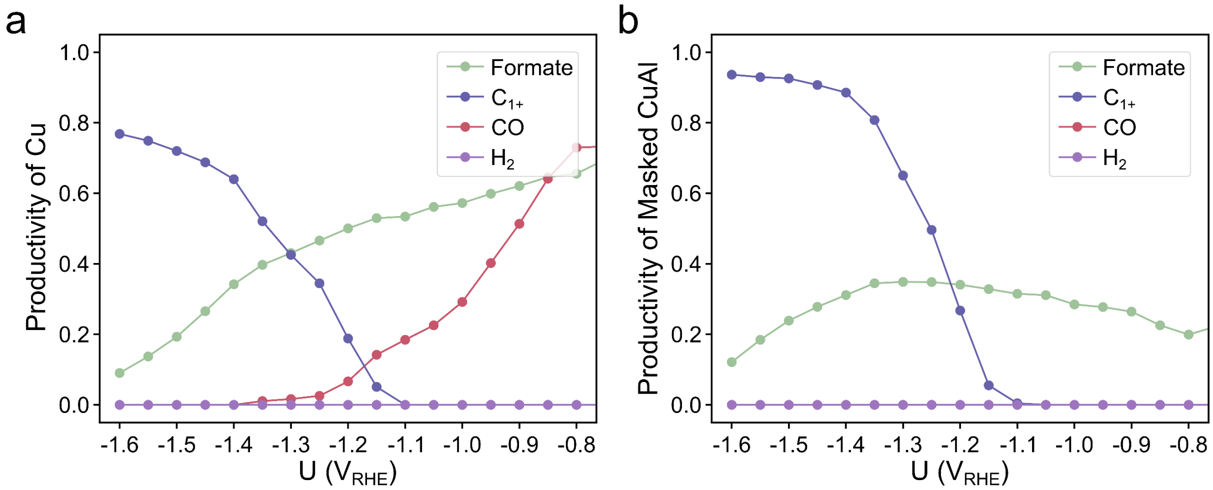

Figure S11. Productivity of (a) pure Cu and (b) masked Cu-Al without normalization. The active site, composition and CN conditions were applied in the case of masked Cu-Al.

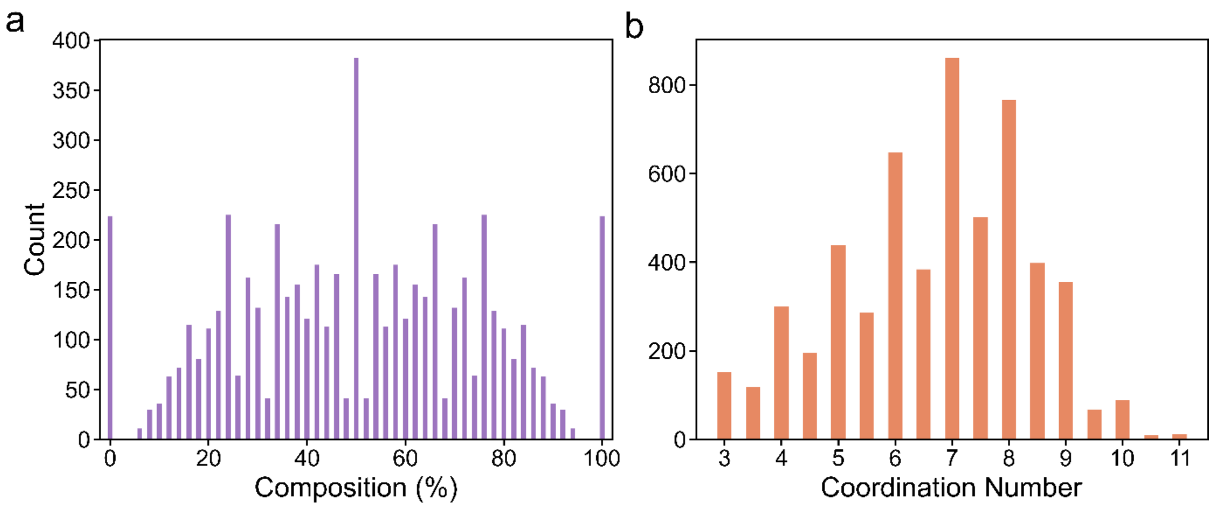

Figure S12. (a) Composition and (b) coordination number distribution of unique active motifs in the dataset.

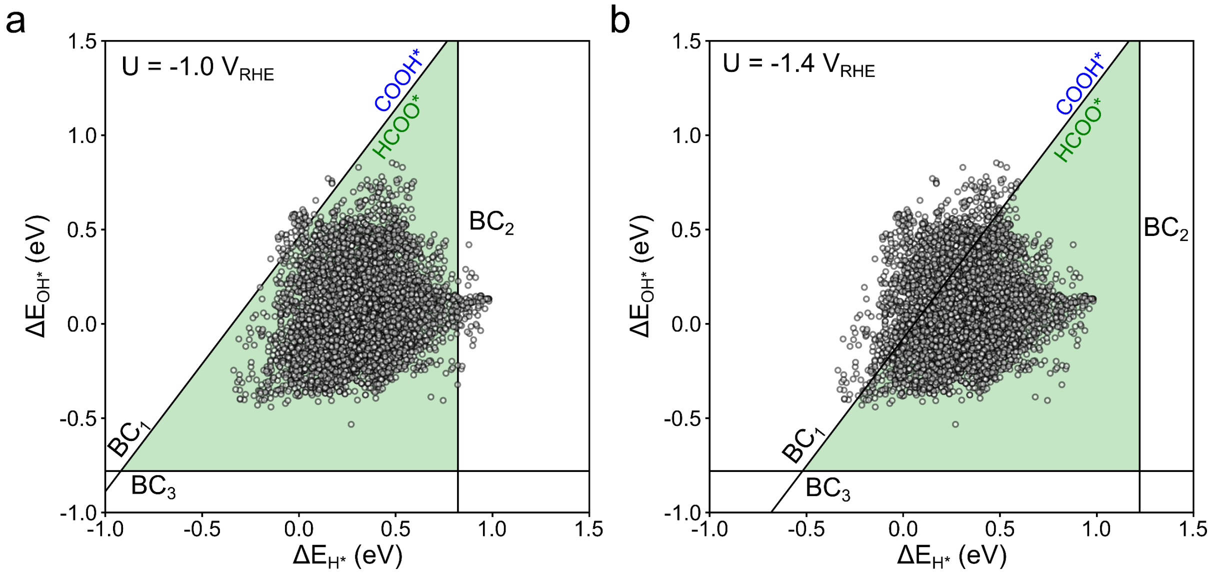

Figure S13. Predicted binding energies of Cu-Ga alloy scattered on 2D selectivity map at two applied potentials of (a) -1.0 V_{RHE} and (b) -1.4 V_{RHE}. Boundary condition 1, 2 and 3 (BC_1, BC_2 and BC_3) are shown. The 2D selectivity map and the boundary conditions are derived using a fixed ΔE_{CO*} value of 0 eV to help intuitive understanding. The area colored as green indicates the region selective for formate. Some of the scattered points, originally located in the formate selective area at U = -1.0 V_{RHE}, move out of the area at U = -1.4 V_{RHE} due to the line shift by the potential change.

Supplementary Note 3: CO_2RR Evaluation on Cu-Ga Electrode

The Cu-Ga catalyst was prepared by one-step electroplating over a polished Cu foil substrate. In brief, the electropolished Cu foil was placed in a solution containing 20 mM gallium sulfate, 10 mM cupric sulfate, 0.5 M potassium pyrophosphate, and 0.1 M nitrilotriacetic acid. The plating bath pH was adjusted to ca. 8.5 using 0.1 M KOH solution. The Cu-Ga film was prepared by electrochemical deposition at -1.6 V vs. Ag/AgCl for 100 s in a stirred above-mentioned electrolyte. The as-plated films were rinsed with copious amounts of deionized water and dried by the nitrogen stream for subsequent measurements.

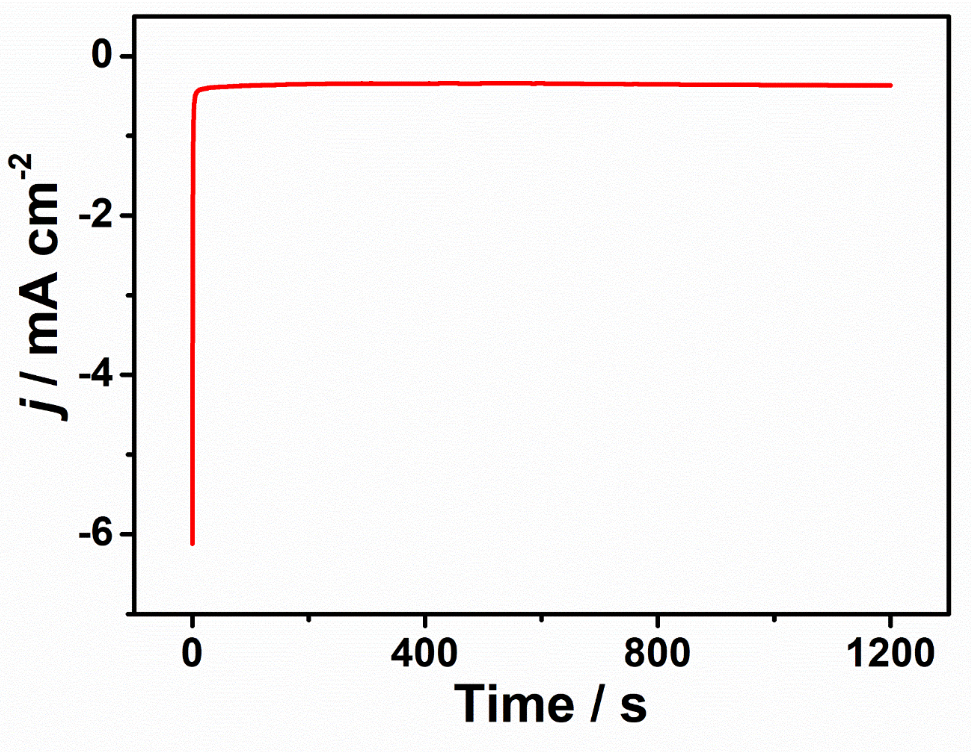

Figure S14. The electrochemical pre-reduction curve of Cu-Ga electrode at a fixed potential of -0.6 V vs. RHE in 0.1 M Ar-saturated $CsHCO_3$ solution for 1200 s.

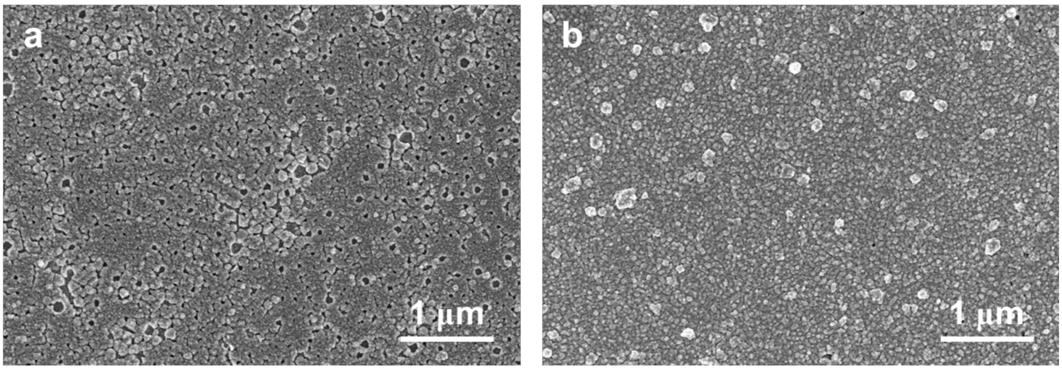

Figure S15. *Ex situ* SEM image of Cu-Ga electrode (a) before and (b) after two hours CO_2 electrolysis. In contrast to the relatively flat surface of polished Cu, the electroplated Cu-Ga electrode consists of densely packed nanoparticles.

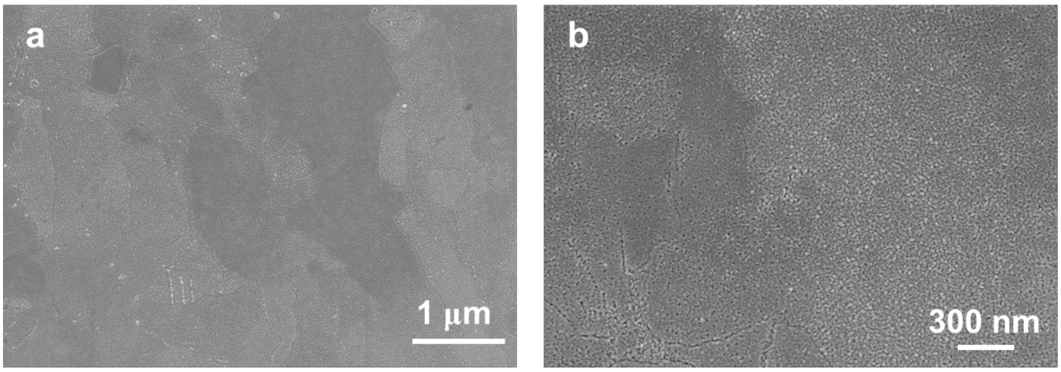

Figure S16. (a) *Ex situ* SEM image of polished Cu foil, (b) enlarged version from panel (a).

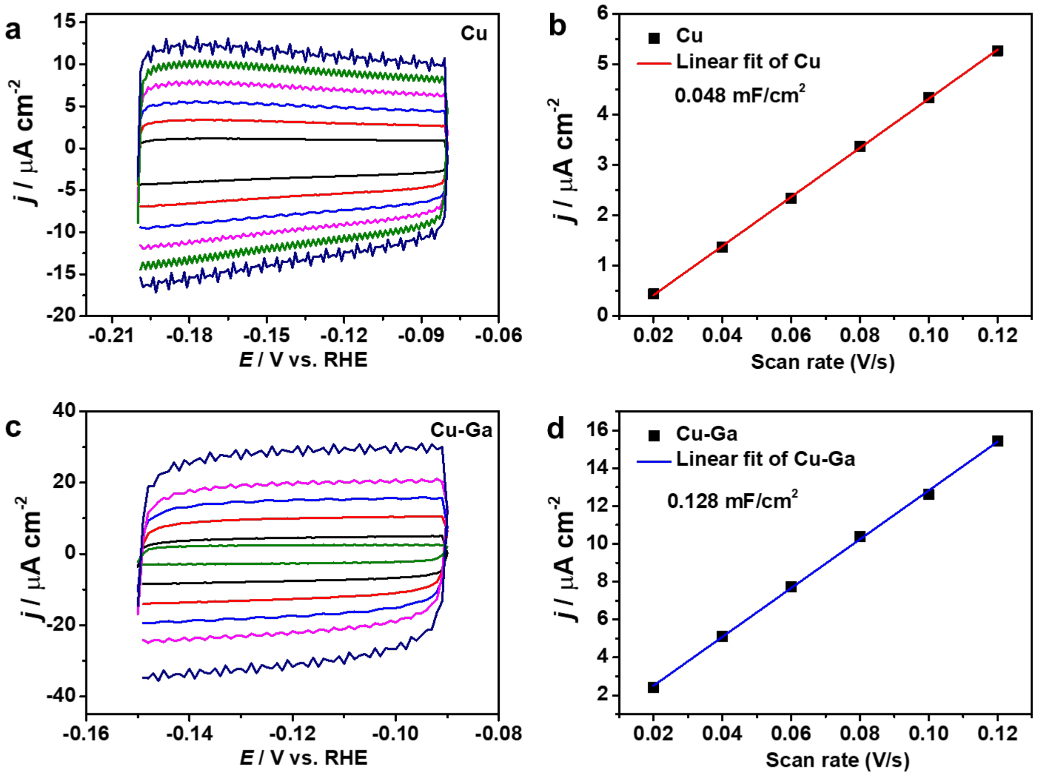

Figure S17. Electrochemical double layer capacitance measurements for (a, b) polished Cu foil and (c, d) Cu-Ga electrode after CO_2RR electrolysis at -0.95 V vs. RHE for 5400 s. The roughness factor of the Cu-Ga electrode is 2.67 times than the polished Cu electrode.

Prior to CO_2RR measurements, the Cu-Ga electrode was subjected to a pre-reduction at -0.6 V in 0.1 M Ar-saturated $CsHCO_3$ for 1200 s toward a stable surface state (**Figure S14**). Typical SEM images in **Figure S15a** and **S15b** depict the morphology of Cu-Ga catalyst before and after CO_2RR electrolysis, respectively. In contrast to the relatively flat surface of polished Cu (**Figure S16**), the electroplated Cu-Ga consists of densely packed nanoparticles, which contributes to the 2.67-times higher surface roughness compared to bare Cu (**Figure S17**).

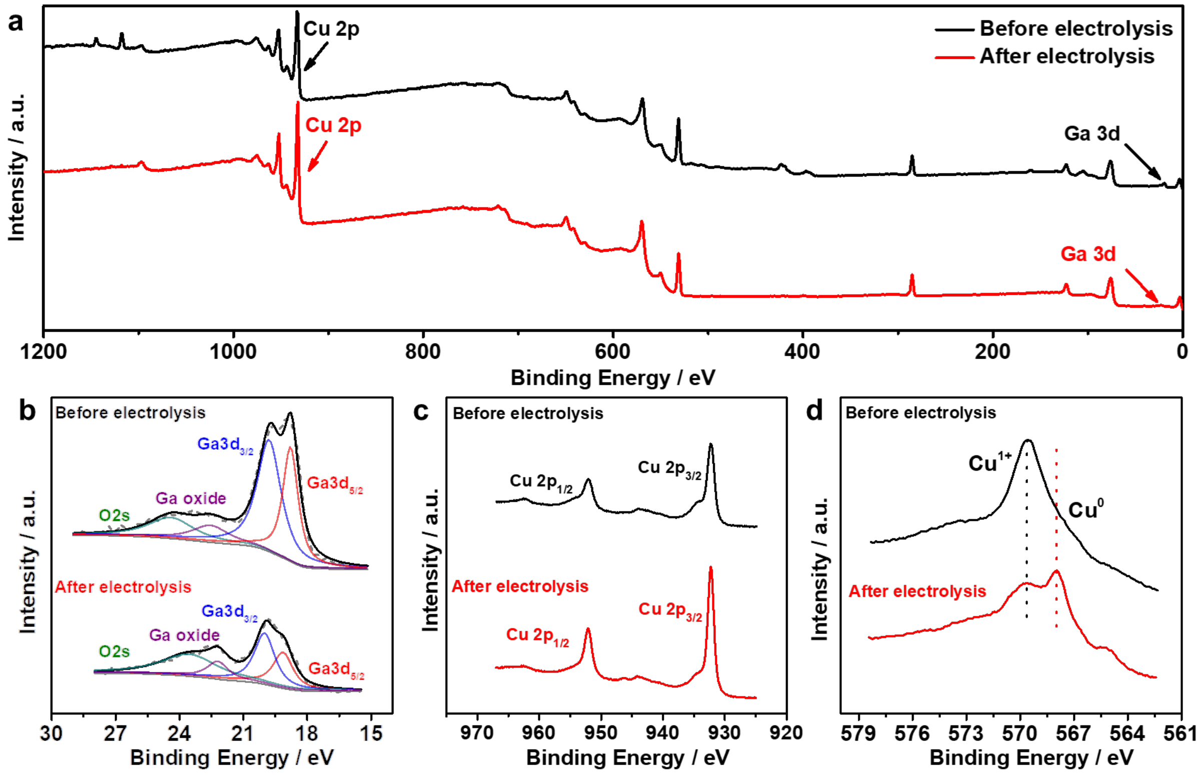

Figure S18. Comparison of the XPS spectra over Cu-Ga electrode before and after CO_2RR electrolysis. (a) XPS survey spectra, (b) core-level Ga 3d spectra overlapped with O 2s, (c) Cu 2p and (d) LMM Auger spectra. Partially oxidized Cu surface species noted in the LMM spectra are probably arisen from the exposure to air during sample transfer process.

X-ray photoelectron spectroscopy (XPS) measurements were then carried out to probe the near-surface componential evolution. As shown in **Figure S18**, only C, O, Cu and Ga signals were observed in the *ex situ* XPS survey spectra, where the atomic ratio of Cu to Ga decreased from 2.1:1 to 5.3:1 after electrochemical reduction (**Table S8**), probably due to the liquid nature of metallic Ga that leaches away from electrode surface into electrolyte under CO_2RR conditions. Noteworthy, this near-surface Ga content is much higher than the bulky ratio as probed by energy dispersive spectroscopy (EDS), suggestive the surface enrichment of Ga in the Cu-Ga electrode (**Figure S19**).

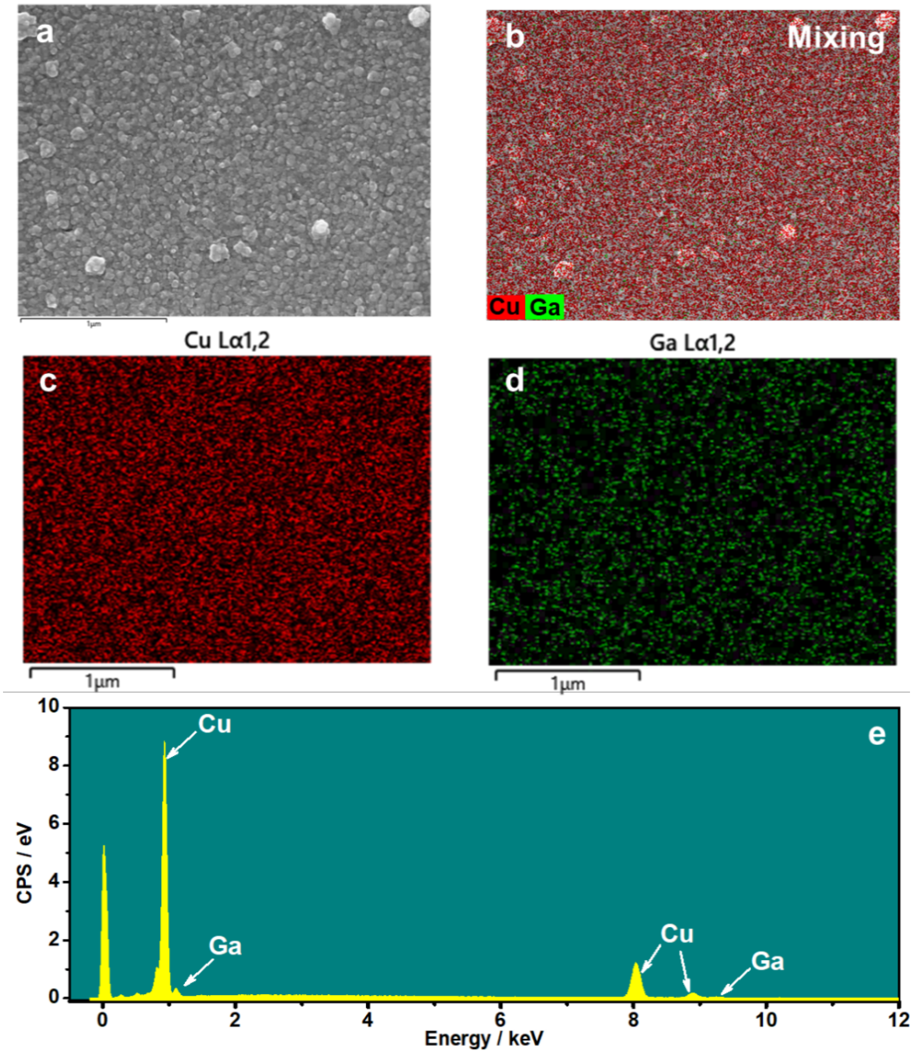

Figure S19. The EDS mapping and spectrum of Cu-Ga electrode after CO_2RR electrolysis. (a) SEM image, (b) Mixing element, (c) Cu element, (d) Ga element, and (e) EDS spectrum. Noteworthy, this near-surface Ga content is much higher than the bulky ratio (**Table S9**) as probed by energy dispersive spectroscopy (EDS), suggestive the surface enrichment of Ga in the Cu-Ga electrode.

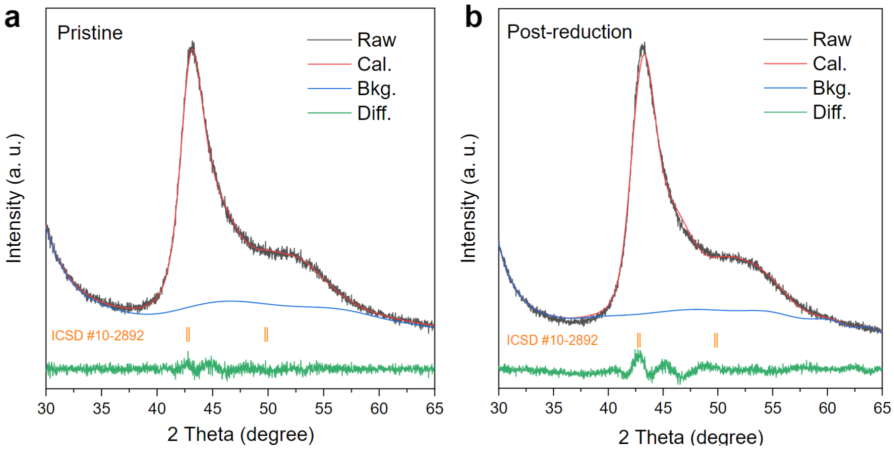

Figure S20. GI-XRD patterns of (a) pristine and (b) spent Cu-Ga electrode after CO_2RR electrolysis. Rietveld refinement has been applied to quantitively probe the crystalline structure (**Table S9**). In line with the reference of $Cu_{0.85}Ga_{0.15}$ ICS D#10-2892, both pristine and spent Cu-Ga electrodes share the same *Fm*-3*m* space group , reinforcing the alloying structure. The spent Cu-Ga electrode exhibits a smaller cell vol ume compared to that for pristine Cu-Ga, as due to the gradually leaching of metallic G a content during CO_2RR and in good harmony with our previous XPS and EDS results.

Grazing-incidence X-ray diffraction (GI-XRD) at α = 0.5° has been carried to better characterize the alloying structure for the Cu-Ga electrodes before and post CO_2RR electrolysis. As shown in **Figure S20**, two major peaks at 42.7° and 49.8° are noted for both the pristine and spent Cu-Ga electrodes deposited on glassy carbon substrates, corresponding to the (111) and (200) diffraction features of $Cu_{0.85}Ga_{0.15}$ (ICSD#10-2892) alloy, respectively. Additionally, bo th pristine and spent Cu-Ga electrodes share the same *Fm*-3*m* space group, reinforcing th e alloying structure. The spent Cu-Ga electrode exhibits a smaller cell volume compared to that for pristine Cu-Ga (**Table S9**), as due to the gradually leaching of metallic Ga co ntent during CO_2RR and in good harmony with our previous XPS and EDS results.

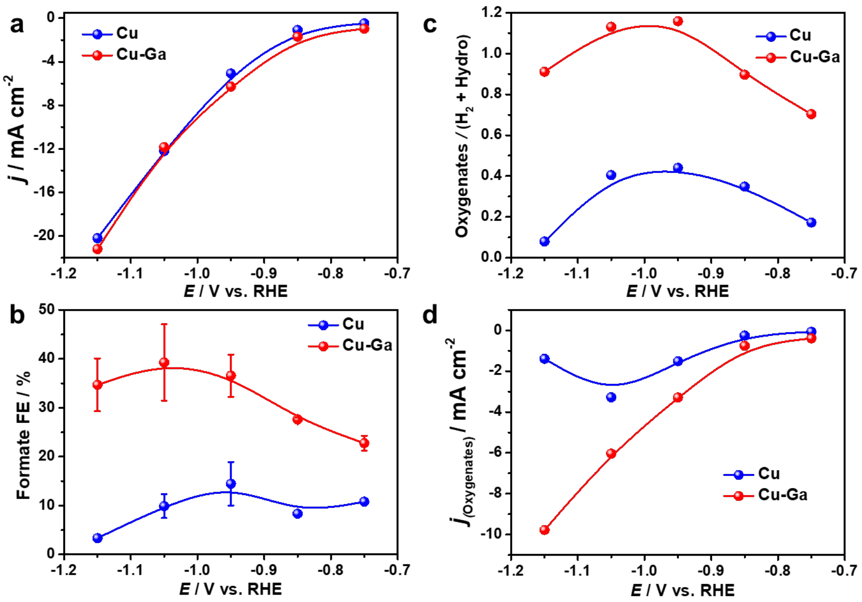

Figure S21. (a) Steady-state current densities, together with (b) the Faradaic efficiency of formate, (c) the selectivity ratio of oxygenates to the sum of H_2 and hydrogenates, and (d) the partial current density of oxygenates on polished Cu versus plated Cu-Ga electrodes under different applied potentials.

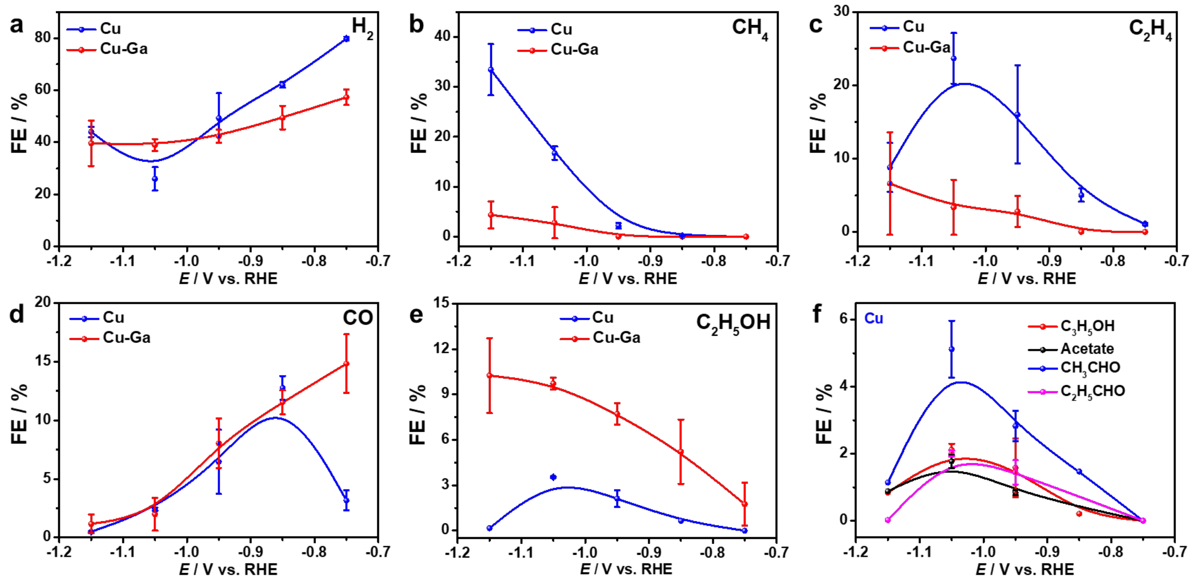

Figure S22. Faradaic efficiency for CO_2 electroreduction products at different applied potential for polished Cu and Cu-Ga electrodes. (a) H_2, (b) CH_4, (c) C_2H_4, (d) CO, (e) C_2H_5OH, (f) other products for polished Cu electrode.

The CO_2RR performance was then screened in 0.1 M CO_2-saturated $CsHCO_3$ electrolyte for polished Cu and plated Cu-Ga, with the gaseous and the liquid products quantitively analyzed by online gas chromatography and ^{1}H nuclear magnetic response (NMR) spectroscopy, respectively. As plotted in **Figure S21**, similar steady-state current densities were noted on those two electrodes throughout the potential window of interest, however, the selectivity of products is quite different (**Figure S22**). A higher H_2 Faradaic efficiency (FE) was observed on Cu over Cu-Ga at small overpotential regime, whereas at more negative potentials below -0.95 V, Cu electrode delivers a lower H_2 FE but significantly higher hydrocarbon generation of CH_4 and C_2H_4 compared to those on Cu-Ga. In contrast, Cu-Ga catalyst favors the liquid oxygenates generation including formate and ethanol. As shown in **Figure S21b**, Cu-Ga delivers a maximum formate FE of ~38.4% at -1.05 V vs. RHE, which is 4 times as high as that on bare Cu. **Figure S21c** plots the ratio of oxygenates to the sum of H_2 and hydrocarbons as a function of cathodic potential, highlighting the promoted oxygenates selectivity on Cu-Ga alloy. Moreover, **Figure S21d** depicts the potential dependence of oxygenates partial current density, in which Cu-Ga delivers ~7 times higher $j_{oxygenates}$ than bare Cu at -1.15 V, experimentally confirming ML-predicted and DFT-calculated results. Considering the mass transport limitation of CO_2 in aqueous solution, we did not continue to measure CO_2RR products distribution below -1.2 V.

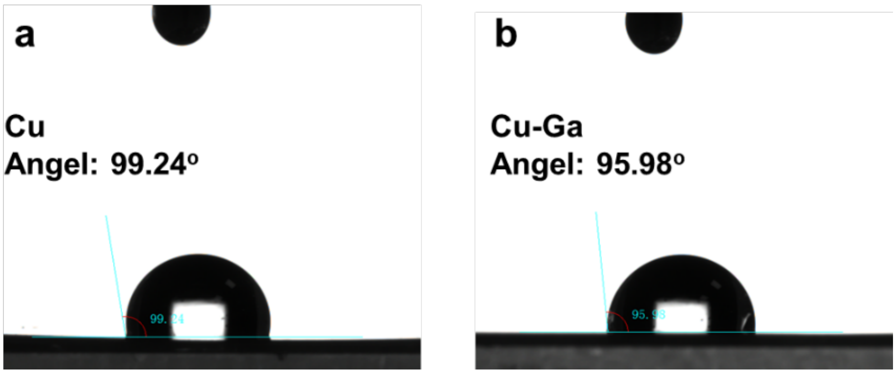

Figure S23. The contact angel of (a) polished Cu and (b) plated Cu-Ga electrodes. Compared to bare Cu, the slightly lower contact angel on plated Cu-Ga electrode suggests its enhanced surface hydrophilicity.

Last but not least, we considered the potential surface hydrophilic effect on formate selectivity. As shown in **Figure S23**, a slightly lower contact angel was noted on Cu-Ga compared to bare Cu, suggesting an enhanced surface hydrophilicity of the former which may synergistically contribute to enhance the CO_2-to-formate selectivity arisen from the surface hydride mechanism.[3, 4] Nevertheless, the similar potential dependence of CO selectivity on Cu and Cu-Ga, as well as the difference in products distribution of hydrocarbons and ethanol highlights the dominant effect of Ga-doping in tuning CO_2RR pathways.

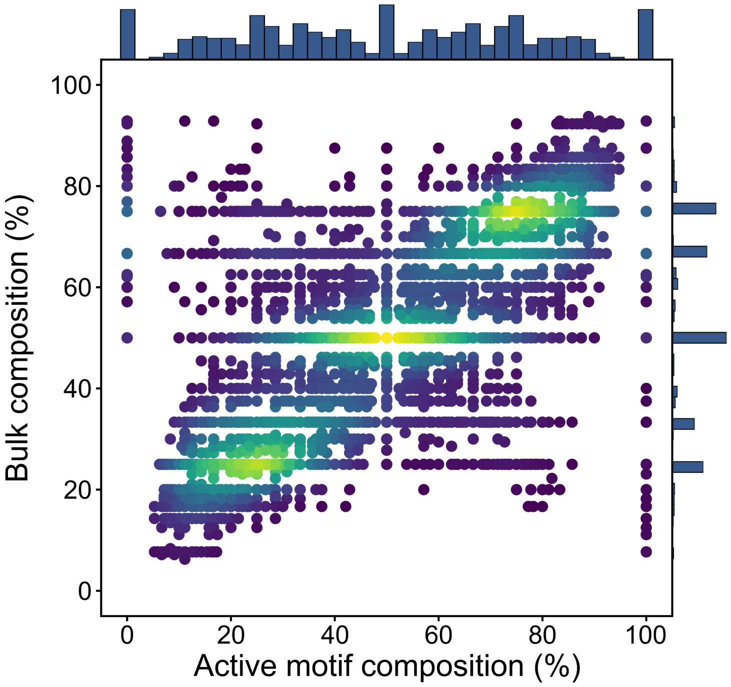

Figure S24. Parity plot of bulk and active motif composition. The lighter color indicates a high density of points.

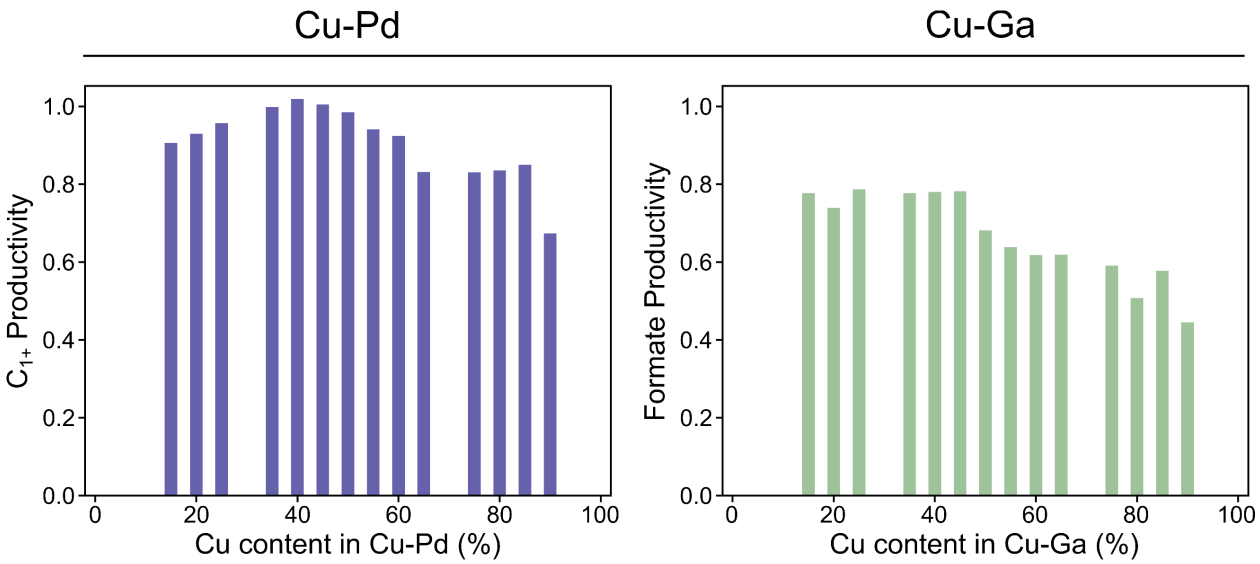

Figure S25. Composition-dependent C_{1+} productivity of Cu-Pd and formate productivity of Cu-Ga at -1.4 V_{RHE} when compositions were derived from bulk structures.

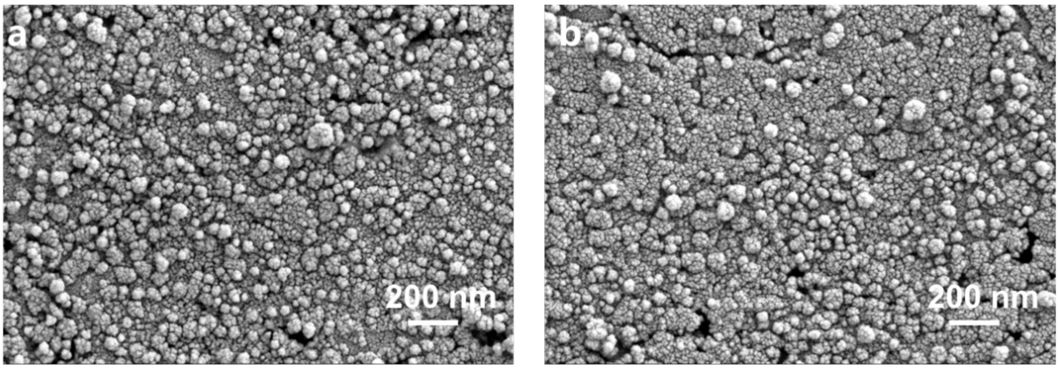

Figure S26. *Ex situ* SEM image of Cu-Pd electrode (a) before and (b) after 2-h CO_2 electrolysis.

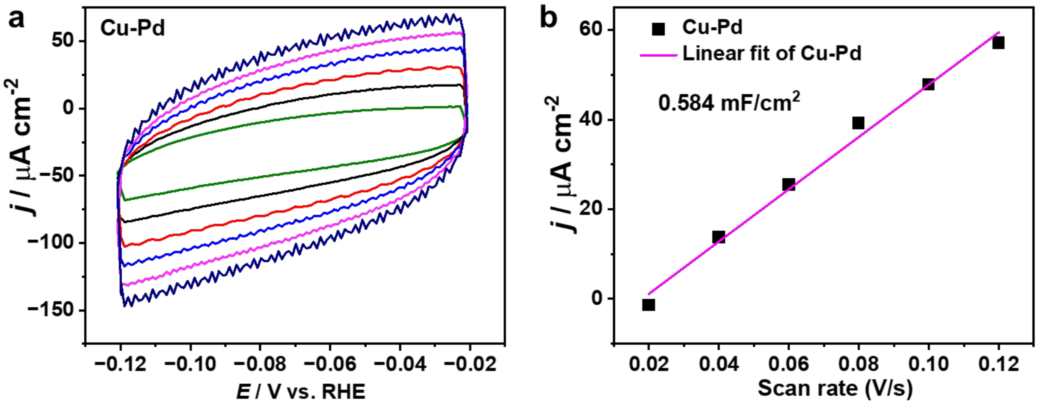

Figure S27. Electrochemical double layer capacitance measurements for (a, b) Cu-Pd electrode. Given the EDLC of 0.048 mF/cm^2 for polished Cu foil (**Figure S22**), the roughness factor of Cu-Pd electrode is 12.16 times as high as that of polished Cu.

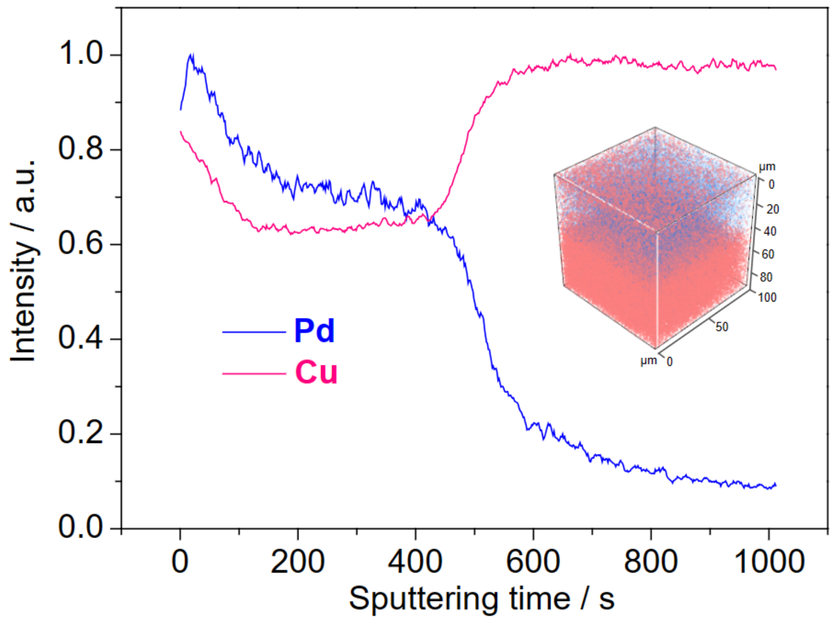

Figure S28. Depth profile of TOF-SIMS measurement on as-prepared Cu-Pd electrode. Insert shows the overlapped distribution of Cu (reddish) and Pd (blue) components.

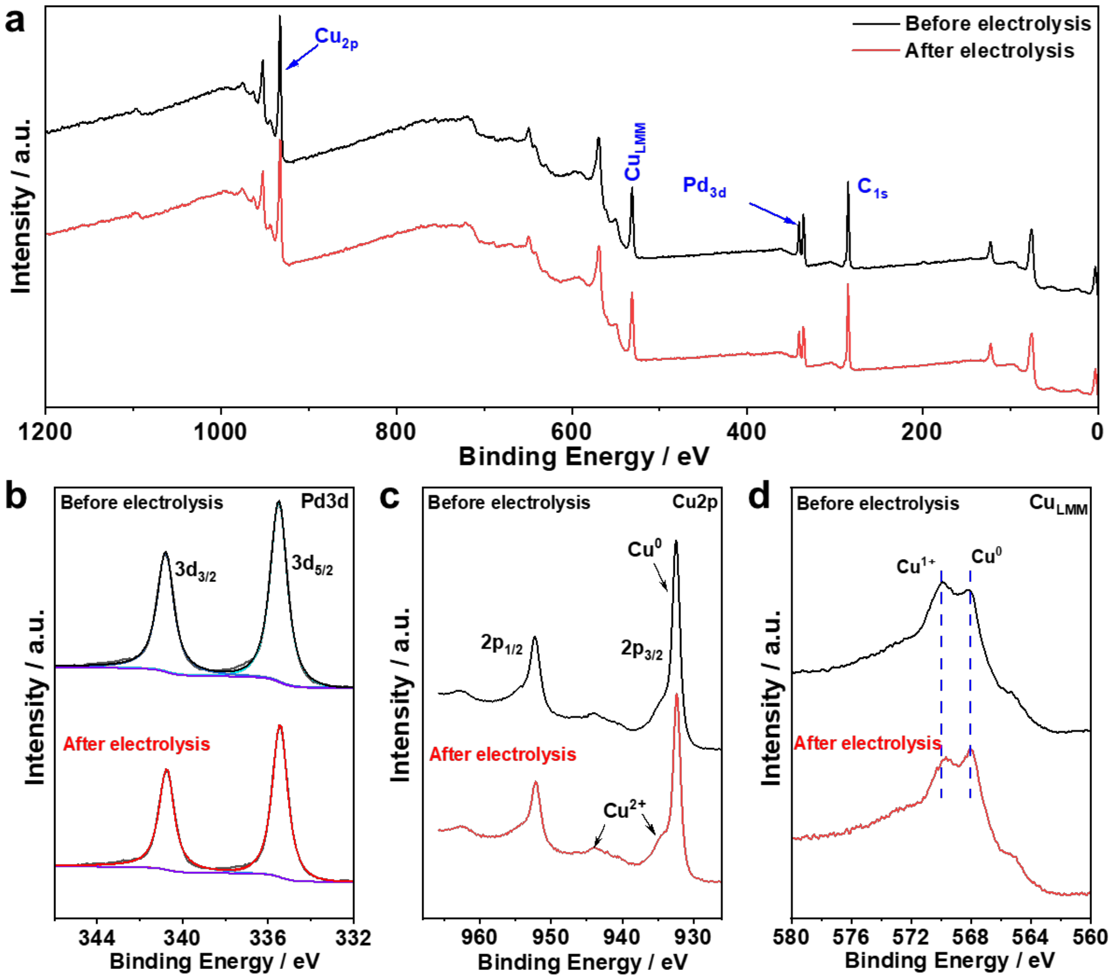

Figure S29. Comparison of the XPS spectra over Cu-Pd electrode before and after CO_2RR electrolysis. (a) XPS survey spectra, (b) core-level Pd 3d spectra, (c) Cu 2p and (d) LMM Auger spectra. Partially oxidized Cu surface species noted in the LMM spectra probably arose from the exposure to air during sample transfer process. The near surface ratio of Cu:Pd slightly increases from 4:1 for pristine electrode to ~ 4.5:1 after CO_2RR electrolysis, suggesting a relatively stable feature of Cu-Pd alloy.

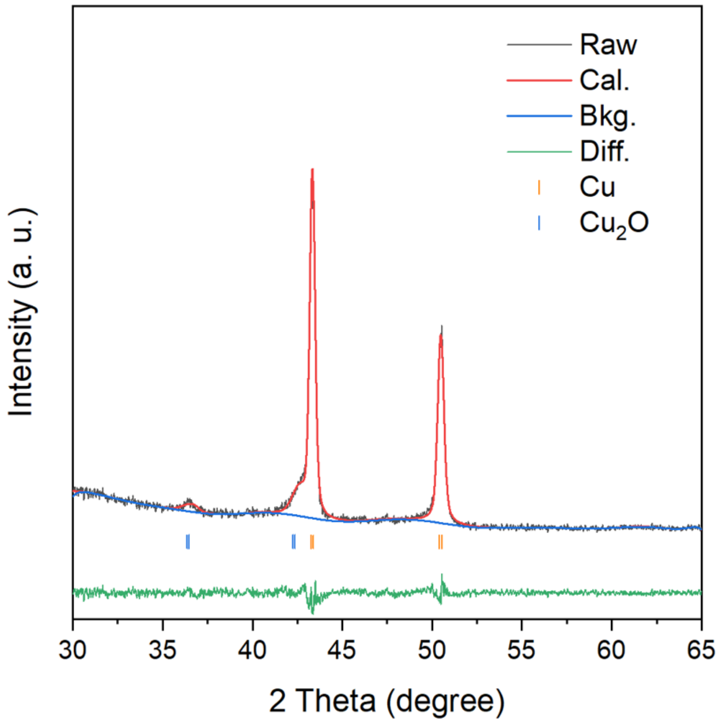

Figure S30. GI-XRD pattern of as-deposited Cu-Pd electrode with Rietveld refinement. Both metallic Cu (ICSD#67-0301) and Cu_2O (ICSD#26-1853) components were observed, but at the absence of Cu-Pd alloy phase.

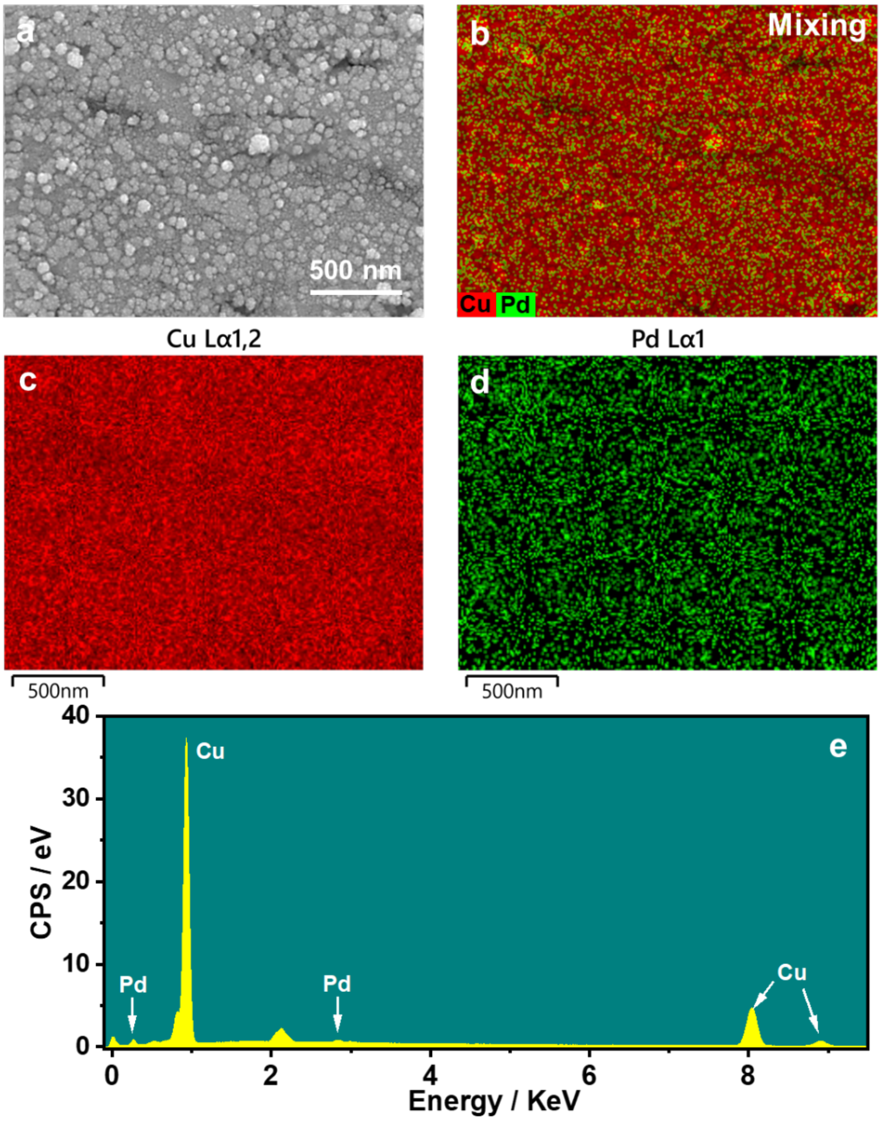

Figure S31. SEM image and relevant EDS mapping for Cu-Pd electrode after CO_2RR. (a) SEM image, (b) mixed element distribution, (c) Cu and (d) Pd element mapping, and (e) EDS spectrum.

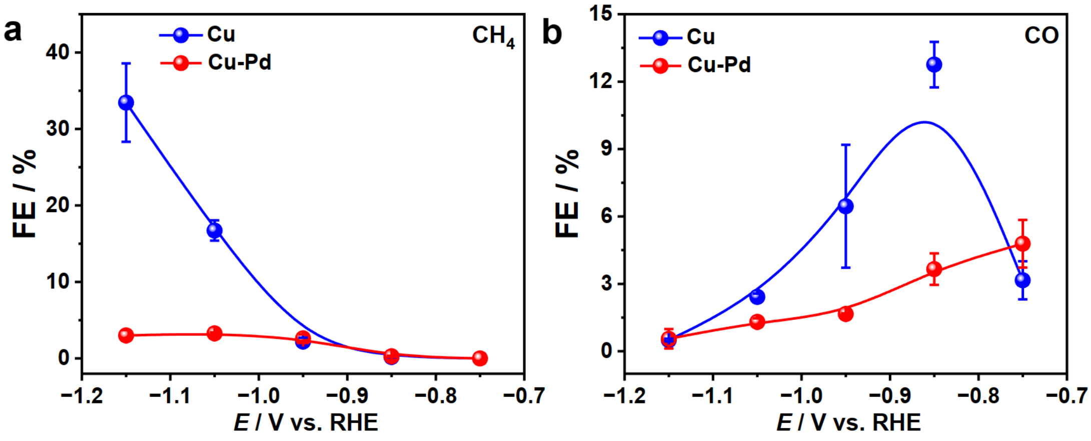

Figure S32. Faradaic efficiency for CO_2 electroreduction gas products at different applied potential for polished Cu and Cu-Pd electrodes. (a) CH_4 (b) CO.

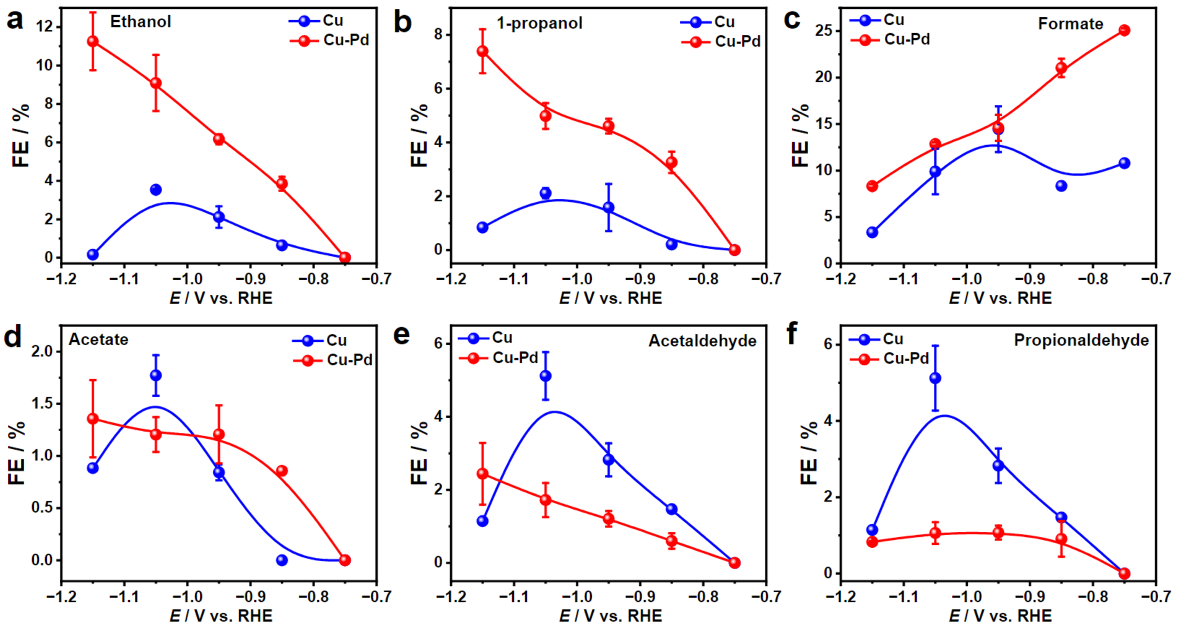

Figure S33. Faradaic efficiency for CO_2 electroreduction liquid products at different applied potential for polished Cu and Cu-Pd electrodes. (a) ethanol, (b) 1-propanol, (c) formate, (d) acetate, (e) acetaldehyde, (f) propionaldehyde.

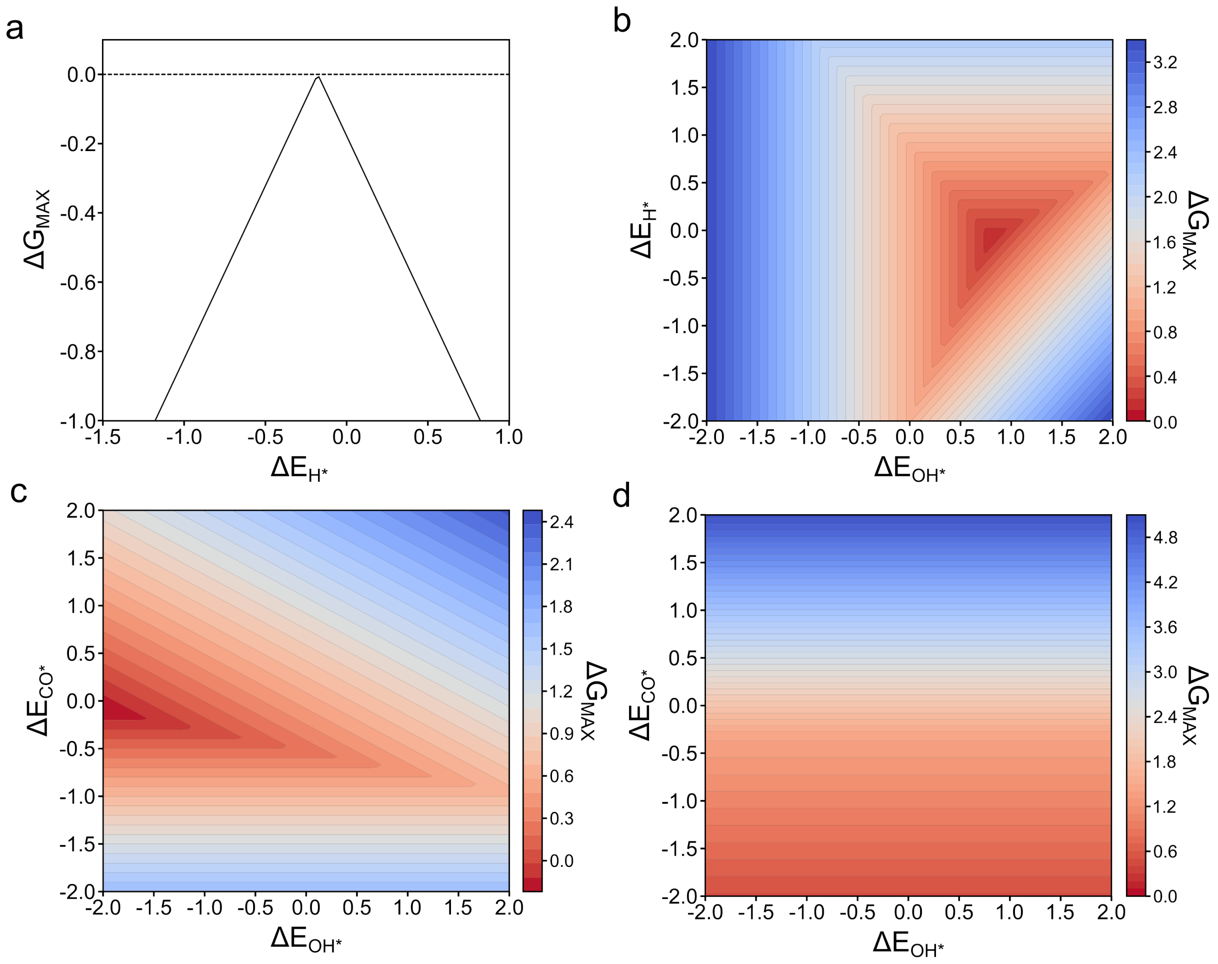

Figure S34. One and two-dimensional diagrams to illustrate how the maximum reaction energy (ΔG_{MAX}) for (a) H_2, (b) formate, (c) CO and (d) C_{1+} production changes in terms of three binding energy descriptors at 0 V_{RHE} of the applied potential.

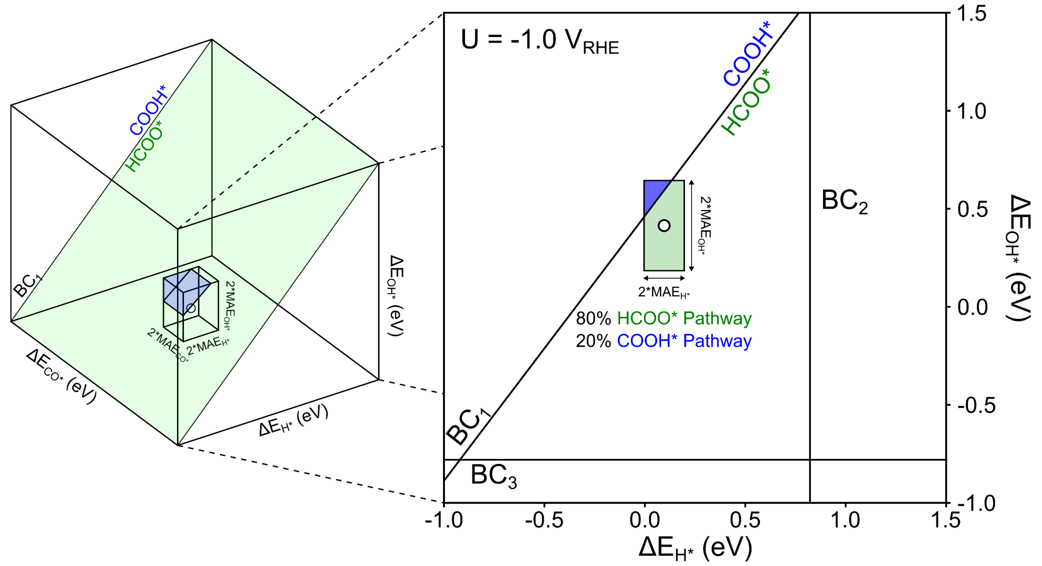

Figure S35. An example of uncertainty contribution for a point near the boundary condition in the 2D projections (right) of 3D selectivity map (left). The uncertainty caused by the ML prediction is represented as a volume based on the MAE of each ML model. Since the volume intersects with the boundary condition, the active motif also contributes to the productivity of the product from another pathway (COOH*) along with the predicted pathway (HCOO*).

Table S1. The mean absolute errors (eV) of each split of 5-fold cross validation.

	Split 1	Split 2	Split 3	Split 4	Split 5	Avg.	Std.
CO	0.121	0.118	0.118	0.115	0.119	0.119	0.002
H	0.105	0.104	0.110	0.109	0.105	0.107	0.003
OH	0.241	0.227	0.220	0.218	0.231	0.227	0.009

Table S2. Top 20 active and selective elemental combination and corresponding productivity for each product.

	Formate Selective		CO Selective		C_{1+} Selective		H_2 Selective	
	Alloy	Productivity	Alloy	Productivity	Alloy	Productivity	Alloy	Productivity
1	In-Sn	1.000	Ag-Ag	1.000	Cu-Pd	1.000	Mn-Ru	1.000
2	Pb-Sn	0.996	Ag-Au	0.929	Mn-Pd	0.784	Rh-Ru	0.974
3	Pb-Zn	0.985	Ag-Sb	0.901	Cr-Pd	0.770	Mn-Rh	0.970
4	Ag-Sn	0.982	Au-Au	0.879	Cu-Ni	0.715	Co-Ru	0.969
5	In-Pb	0.971	Au-Sb	0.862	Au-Pd	0.691	Co-Mn	0.963
6	Sb-Sn	0.969	Au-Ge	0.788	Cu-Pt	0.687	Ni-Ru	0.959
7	Ag-In	0.955	Au-Pb	0.688	Ni-Pd	0.665	Co-Rh	0.957
8	Ga-Sn	0.953	Au-Sn	0.679	Pd-Pd	0.647	Fe-Ru	0.935
9	In-In	0.952	Ag-Pb	0.616	Cu-Cu	0.640	Co-Ni	0.932
10	Sn-Zn	0.941	Au-Zn	0.613	Cr-Pt	0.638	Co-Co	0.931
11	In-Sb	0.937	Au-In	0.607	Pd-Ti	0.629	Fe-Rh	0.928
12	Ge-Pb	0.934	Ag-As	0.593	Mn-Pt	0.628	Fe-Mn	0.926
13	Ag-Ga	0.931	Ni-Se	0.535	Pd-Re	0.624	Ru-Ru	0.919
14	Sb-Zn	0.923	Ag-Sn	0.530	Pd-V	0.623	Mn-Ni	0.918
15	In-Zn	0.912	As-Se	0.514	Pt-Pt	0.618	Ni-Rh	0.915
16	Ag-Zn	0.907	Au-Se	0.510	Cr-Cu	0.615	Mn-Re	0.906
17	Ag-Pb	0.905	Au-Ga	0.502	Ag-Pd	0.602	Mn-Os	0.899
18	Ga-Pb	0.903	Al-Au	0.484	Pt-Si	0.584	Ir-Mn	0.895
19	Ge-In	0.903	Co-Se	0.479	Pd-Rh	0.582	Co-Ir	0.888
20	Pb-Pb	0.897	Ag-Se	0.477	Pt-Ti	0.581	Co-Fe	0.885

Table S3. The equation of boundary conditions expressed using binding energy descriptors and the applied potentials.

Boundary Condition	Expression
$\Delta G_{rxn}^{(i)} = \Delta G_{rxn}^{(ii)}$	$0.601\ \Delta E_{CO*} - 0.740\ \Delta E_{OH*} + \Delta E_{H*} + 1.343 + eU = 0$
$\Delta G_{rxn}^{(iii)} = 0$	$\Delta E_{H*} + 0.178 + eU = 0$
$\Delta G_{rxn}^{(iv)} = 0$	$\Delta E_{OH*} - 0.22 - eU = 0$
$\Delta G_{rxn}^{(v)} = 0.75$	$0.563\ \Delta E_{CO*} + 0.857 + eU = 0\ (\Delta E_{CO*} + 0.32 < 0)$<br>$1.563\ \Delta E_{CO*} + 1.222 + eU = 0\ (\Delta E_{CO*} + 0.32 \geq 0)$
$\Delta G_{rxn}^{(vi)} = 0$	$0.507\ \Delta E_{CO*} + \Delta E_{H*} + 0.89 = 0$
$\Delta G_{rxn}^{(vii)} = 0$	$\Delta E_{H*} + 0.178 - eU = 0$

Table S4. The atomic contents distribution in Cu-Pd electrode prior to and after electrolysis.

Electroplated Cu-Pd		Cu at.%	Pd at.%	Cu : Pd
before electrolysis	XPS	79.95	20.05	3.98:1
	EDS	98.76	1.24	79.64:1
after electrolysis	XPS	81.94	18.06	4.53:1
	EDS	99.00	1.00	99:1

Table S5. The Gibbs free energy correction values for gaseous molecules. For all calculations, temperature was set to 298.15 K.

Species	Fugacity (Pa)	E_{zpe} (eV)	$\int C_p\, d$ (eV)	$-TS$ (eV)	E_{gas} (eV)	G_{corr} (eV)
H_2O	3,534	0.572	0.110	– 0.676	0.010	0.016
H_2	101,325	0.269	0.090	– 0.402	-	– 0.043
CO_2	101,325	0.304	0.098	– 0.662	0.460	0.200
CO	101,325	0.130	0.090	– 0.610	0.070	– 0.320
HCOOH	101,325	0.885	0.124	– 0.830	0.270	0.449

Table S6. The Gibbs free energy correction values for adsorbates.

Species	E_{zpe} (eV)	$\int C_p\, d$ (eV)	$-TS$ (eV)	E_{solv} (eV)	G_{corr} (eV)
H*	0.160	0.005	– 0.007	-	0.158
CO*	0.179	0.085	– 0.230	– 0.100	– 0.066
COOH*	0.567	0.103	– 0.270	– 0.250	0.150
HCOO*	0.583	0.097	– 0.220	-	– 0.460
COH*	0.472	0.080	– 0.151	– 0.250	0.151
CH*	0.368	0.025	– 0.038	-	0.347
CH_2*	0.651	0.042	-0.069	-	0.624

Table S7. The hyperparameters of ML regression algorithms determined by Bayesian optimization.

Model	Hyperparameters
Gradient Boost Regressor	n_estimators = 4000, learning_rate = 0.15, max_depth =17, min_samples_leaf = 28, min_samples_split = 24

Table S8. The atomic contents distribution in Cu-Ga electrode prior to and after electrolysis.

Electroplated Cu-Ga		Cu at.%	Ga at.%	Cu : Ga
before electrolysis (XPS)		67.2	32.8	2.1:1
after electrolysis	XPS	84.1	15.9	5.3:1
	EDS	92.0	8.0	11.9:1

Table S9. Structural information, including space group, lattice parameters, occupancy, discrepancy factor (R_{wp}), and goodness fit factor (χ^2), derived from the Rietveld refinement of XRD spectra and $Cu_{0.85}Ga_{0.15}$ (ICSD :102892) standards.

Sample	Space group	Cell parameter	Occupancy	Cell volume	R_{wp}	χ^2
$Cu_{0.85}Ga_{0.15}$ (ICSD#10-2892)	*Fm*-3*m*	a: 3.6573<br>b: 3.6573<br>c: 3.6573	Cu: 0.850<br>Ga: 0.150	48.919	-	-
Pristine Cu-Ga		a: 3.6620<br>b: 3.6620<br>c: 3.6620	Cu: 0.727<br>Ga: 0.273	49.108	0.021	1.293
Spent Cu-Ga		a: 3.6515<br>b: 3.6515<br>c: 3.6515	Cu: 0.876<br>Ga: 0.124	48.687	0.031	2.563

Supplementary References

1. Tang, M. T.; Peng, H.; Lamoureux, P. S.; Bajdich, M.; Abild-Pedersen, F., From electricity to fuels: descriptors for C_1 selectivity in electrochemical CO_2 reduction. *Appl. Catal. B: Environ.* **2020,** *279*, 119384.

2. Liu, X.; Xiao, J.; Peng, H.; Hong, X.; Chan, K.; Nørskov, J. K., Understanding trends in electrochemical carbon dioxide reduction rates. *Nat. Commun.* **2017,** *8* (1), 15438.

3. Ye, C.; Raaijman, S. J.; Chen, X.; Koper, M. T., Enhanced electrochemical CO_2 reduction to formate on poly (4-vinylpyridine)-modified copper and gold electrodes. *ACS Appl. Mater. Interfaces* **2022,** *14* (40), 45263-45271.

4. Buckley, A. K.; Lee, M.; Cheng, T.; Kazantsev, R. V.; Larson, D. M.; Goddard III, W. A.; Toste, F. D.; Toma, F. M., Electrocatalysis at organic–metal interfaces: identification of structure–reactivity relationships for CO_2 reduction at modified Cu surfaces. *J. Am. Chem. Soc.* **2019,** *141* (18), 7355-7364.
